# Synergistic Interactions in Microbial Biofilms Facilitate the Establishment of Opportunistic Pathogenic Fungi in Household Dishwashers

**DOI:** 10.3389/fmicb.2018.00021

**Published:** 2018-01-30

**Authors:** Jerneja Zupančič, Prem K. Raghupathi, Kurt Houf, Mette Burmølle, Søren J. Sørensen, Nina Gunde-Cimerman

**Affiliations:** ^1^Department of Biology, Biotechnical Faculty, University of Ljubljana, Ljubljana, Slovenia; ^2^Molecular Microbial Ecology Group, Section of Microbiology, Department of Biology, University of Copenhagen, Copenhagen, Denmark; ^3^Laboratory of Hygiene and Technology, Department of Veterinary Public Health and Food Safety, Faculty of Veterinary Medicine, Ghent University, Ghent, Belgium

**Keywords:** synergism, biofilm formation, EPDM, *Exophiala dermatitidis*, dishwashers, multispecies biofilm

## Abstract

Biofilms formed on rubber seals in dishwashers harbor diverse microbiota. In this study, we focussed on the microbial composition of bacteria and fungi, isolated from a defined area of one square centimeter of rubber from four domestic dishwashers and assessed their abilities to *in vitro* multispecies biofilm formation. A total of 80 isolates (64 bacterial and 16 fungal) were analyzed. Multiple combinations of bacterial isolates from each dishwasher were screened for synergistic interactions. 32 out of 140 tested (23%) four-species bacterial combinations displayed consistent synergism leading to an overall increase in biomass, in all experimental trails. Bacterial isolates from two of the four dishwashers generated a high number of synergistically interacting four-species consortia. Network based correlation analyses also showed higher co-occurrence patterns observed between bacterial members in the same two dishwasher samples, indicating cooperative effects. Furthermore, two synergistic four-species bacterial consortia were tested for their abilities to incorporate an opportunistic fungal pathogen, *Exophiala dermatitidis* and their establishment as biofilms on sterile ethylene propylene diene monomer M-class (EPDM) rubber and polypropylene (PP) surfaces. When the bacterial consortia included *E. dermatitidis*, the overall cell numbers of both bacteria and fungi increased and a substantial increase in biofilm biomass was observed. These results indicate a novel phenomenon of cross kingdom synergy in biofilm formation and these observations could have potential implications for human health.

## Introduction

Biofilms are defined as highly structured communities of microorganisms that are attached to each other, commonly surface associated and enclosed within a self-produced matrix of extracellular polymeric substance (EPS) (Costerton et al., 1995). The advantages obtained by organisms from producing biofilms include protection from harsh environments, enhanced tolerance to physical and chemical stress, metabolic cooperation and community-coordinated adjustment of gene expression. Microorganisms in biofilms adapt their physiology and stress responses and display collective and coordinated behavior (Donlan, 2002; Chmielewski and Frank, 2004; Van Houdt and Michiels, 2010).

Multispecies biofilms are common and often dominant in natural environments (Donlan, 2002; Hall-Stoodley et al., 2004). Resident microorganisms interact with each other in both synergistic and antagonistic manner affecting the biofilm biomass, functionality and tolerance compared to mono-species biofilms (Filoche et al., 2004; Sharma et al., 2005; Burmølle et al., 2006; Moons et al., 2009; Wen et al., 2010; Pathak et al., 2012; Schwering et al., 2013; Lee et al., 2014; Ren et al., 2015; Madsen et al., 2016).

Biofilms are a source of food contamination and food safety related problems (Carpentier and Chassaing, 2004; Srey et al., 2013; Røder et al., 2015). In food production facilities, pathogenic bacteria may benefit from biofilm formation (Klayman et al., 2009) as biofilms can withstand higher temperatures, standard cleaning procedures (Marouani-Gadri et al., 2009) and commonly used disinfectants (Corcoran et al., 2014) thereby, leading to biofilm related outbreaks (Donlan, 2002; de Souza et al., 2015). Most studies focus on the biology and persistence of monocultures of a particular bacterial pathogen in biofilm (Lister and Horswill, 2014; Tolker-Nielsen, 2014), however, there is a growing need to understand the impact of interspecies interactions on the formation and architecture of biofilms (Elias and Banin, 2012; Sheppard and Howell, 2016). Increasing evidence points to the role of fungi in biofilms involved in human diseases (Ramage et al., 2009; Hoarau et al., 2016; Kalan et al., 2016). In mixed bacterial and fungal biofilms, it was reported that bacterial cells gained protection within the matrix and increased its tolerance to antimicrobials and stress (De Brucker et al., 2015; Kong et al., 2016).

Recently, it was discovered that the extreme depauperate ecosystem of household appliances, such as dishwashers, washing machines and coffee machines, harbor selected poly-extremotolerant bacteria and fungi (Zalar et al., 2011; Babič et al., 2015; Callewaert et al., 2015; Vilanova et al., 2015; Zupančič et al., 2016; Raghupathi et al., 2018). These microbes resist both high and low pH, temporary increase in temperatures up to 74°C, desiccation, high organic loads, high concentrations of NaCl and mechanical stress from water ejectors (Zalar et al., 2011; Zupančič et al., 2016). They are represented by diverse human opportunistic fungi (Zalar et al., 2011; Döğen et al., 2013; Gümral et al., 2016; Zupančič et al., 2016) and bacteria (Raghupathi et al., 2018).

We have focussed on mixed biofilms in dishwashers since there is a worldwide increase in demand for household appliances (Freedonia, 2016) and opportunistic pathogens detected in these machines could be an emerging threat to human health (Binder et al., 1999; Morens et al., 2004). Despite the ubiquity of microbial communities and the presence of dishwashers in many private households, interspecies interactions among different bacteria and fungi have not been investigated in these systems. The focus of present research was to identify the species composition of bacteria and fungi from the rubber seals of four different dishwashers. The viable bacterial and fungal isolates were identified using a combination of classical and molecular methods. Multiple combinations of different bacterial isolates from each these dishwashers were co-cultured *in vitro* and their ability to form stable, four-species biofilms was assessed. The synergistic bacterial consortia were tested for their ability to incorporate *Exophiala dermatitidis* (the most common opportunistic fungal pathogen found in dishwashers) (Zalar et al., 2011; Döğen et al., 2013; Gümral et al., 2016; Zupančič et al., 2016) and their establishment as mixed bacterial-fungal biofilm on different surfaces commonly used in dishwashers were investigated.

## Materials and Methods

### Cultivation and Identification of Microbial Community

Microbial biofilms formed on 1 cm^2^ area of rubber seal from four different dishwashers were sampled in this study (**Table 1**). The dishwashers varied in age, i.e., years in operation; frequency of use, i.e., the number of times the dishwasher was used per week; and incoming tap water hardness. The water supply connected to these dishwashers (DWs) was characterized based on ion analysis method (Babič et al., 2013). Final concentrations were determined following the method from ISO Standard SIST EN ISO 11885:2009. Biofilm samples were collected with sterile swabs (Invasive sterile EUROTUBO^®^ collection swab). Sampling of microbiota was performed by rubbing a cotton swab moistened with physiological saline over 1 cm^2^ rubber seal surfaces, immediately after the termination of the washing cycle in these dishwashers. Swab samples were stored in sterile collection tubes at 4°C and were processed within a day.

**Table 1:** Dishwashers sampled for microbial composition in this study.

Dishwasher	Country; city; GPRS coordinates	Age (years in use)	Frequency of use/week	Influent water	NCBI SRR
DW1	SI; Žalec; 46°15′3.59″N 15°9′50.18″E	3	7	SH	3279031
DW2	SI; Ljubljana; 46°03″N 14°30″E	5	3	MH	3335242
DW3	SI; Brezovica; 45°58′11.68″N 14°26′9.95″E	7	3	MH	3343759
DW4	SI; Novo Mesto; 45°47′54.88″N 15°10′26.08″E	8	7	MS	3335236


*The dishwashers varied in age, frequency of use and influent water hardness characteristics. DW1, dishwasher 1; DW2, dishwasher 2; DW3, dishwasher 3; DW4, dishwasher 4; SH, slightly hard (1–1.5 mmol/L CaCO_*3*_); MH, moderately hard (1.5 – 2.0 mmol/L CaCO_*3*_); MS, moderately soft (0.5–1.0 mmol/L CaCO_*3*_). ‘SRR’ represents the sequence read archive assigned after deposition of 16s rRNA gene marker-based amplicon reads to NCBI database.*

Viable microbes living in close contact from each of these dishwashers were cultivated by plating methods to obtain individual bacterial and fungal colonies. For each dishwasher sample, 3 ml of sterile physiological saline was added into the collection tube containing swabs and vortexed intensely for 1 min at maximum speed. Subsequently, for bacterial screening, aliquots of 100 μl of the sample were diluted 10-fold and plated on different bacteriological agar media, i.e., nutrient agar (NA), Brain–Heart Infusion agar (BHI), Reasoner’s 2A agar (R2A), and Minimal Media agar (M9) (Vogel and Bonner, 1956). All plates were supplemented with cycloheximide (CYC, 50 μg ml^-1^, Sigma) to ensure only bacterial growth. Plates were incubated aerobically at 37°C for 2 days (NA and BHI) and up to 7 days for M9. In case of R2A, plates were incubated for 7 days at 35°C. Isolation of fungi was performed by inoculating same aliquots of 100 μl of the above diluted suspension on Malt Extract Agar (MEA) (Oxoid, Hampshire, United Kingdom) supplemented with 0.05 g/l chloramphenicol, and incubated at 30 and 37°C for up to 7 days.

Microbial colonies of various morphotypes (both bacterial and fungal) were restreaked several times on chosen media plates Luria Bertani (LB) for bacteria and MEA for fungi until pure cultures were obtained. The pure cultures were deposited and can be obtained from the Ex Culture Collection, part of the Infrastructural Centre Mycosmo (MRICUL) at the Department of Biology, Biotechnical Faculty, University of Ljubljana, Slovenia.

### Identification of Isolates Using Sanger Sequencing

DNA extraction and molecular identification of fungal isolates from dishwashers was performed as previously described (Zupančič et al., 2016). Briefly, pure fungal cultures were transferred to fresh MEA medium and after 3–7 days of incubation, DNA extractions were performed with methods specific to the type of fungal isolates. For yeasts, DNA extraction was done using PrepMan Ultra Sample Preparation Reagent (Applied Biosystems) according to the manufacturer’s instructions. DNA extractions of filamentous fungi and *Exophiala* strains were done according to Gerrits van den Ende and de Hoog (1999), after mechanical lysis of the mycelium. *Fusarium* strains were identified using nuclear translation elongation factor 1-alpha (*tef*) sequences, amplified with the EF1 and EF2 primers (O’Donnell et al., 1998).

Bacterial identification was performed using the extracted genomic DNA from overnight grown pure cultures (LB plates incubated at 37°C) using PrepMan Ultra Sample Preparation Reagent (Applied Biosystems) according to the manufacturer’s instructions. PCR amplifications based on 16S rRNA gene with oligonucleotide primers 27F and 1492R targeting bacterial 16S ribosomal gene (Lane, 1991) were applied for bacterial identification. The amplified fragments were Sanger sequenced (Microsynth AG) and the 16S rRNA gene sequences were trimmed to approx. 800 bp amplicons and identification was done using Ribosomal Database Project-II (RDP)^1^ and National Center for Biotechnology Information (NCBI) BLAST tool searching GenBank. RDP Seqmatch was used against the 16S rRNA database with sequences from isolated bacteria in order to determine the closest known relatives. The sequences were also compared against GenBank non-redundant nucleotide database using NCBI BlastN (Megablast). The isolates were assigned at species level with the Seqmatch score (S-ab) ≥ 0.99 (99% similarity) or at genus level with S-ab score of ≥0.95 (95% similarity). Sequences were uploaded to the NCBI database and the accession numbers are provided (**Table 2**).

**Table 2:** List of selected bacterial isolates used in biofilm cultivation experiments.

Isolate source	ID#	Closest relative	^∗^	Strain ID EXF-/EXB L	Accession number of the closest relative	NCBI Accession number
DW1	1	*Pseudomonas aeruginosa*	*P*	EXB L-1125	KR911837	MG597301
	2	*Ochrobactrum pseudintermedium*	*P*	EXB L-1130	KF026284	MG597302
	3	*Klebsiella oxytoca*	*P*	EXB L-1137	CP011636	MG597303
	4	*Stenotrophomonas maltophilia*	*P*	EXB L-1167	KP185140	MG597304
	5	*Enterobacter hormaechei*	*P*	EXB L-1135	KP303395	MG597305
	6	*Pseudomonas putida*	*P*	EXB L-1149	KJ735915	MG597306
	7	*Bacillus cereus*	*F*	EXB L-1175	KC969074	MG597307

DW2	8	*Acinetobacter lwoffii*	*P*	EXB L-1215	LN774665	MG597308
	9	*Bacillus cereus*	*F*	EXB L-1223	KP988025	MG597309
	10	*Exiguobacterium aestuarii*	*F*	EXB L-1196	FJ462716	MG597310
	11	*Exiguobacterium panipatensis*	*F*	EXB L-1201	EF519705	MG597311
	12	*Kocuria rhizophila*	*A*	EXB L-1199	AY030315	MG597312
	13	*Micrococcus luteus*	*A*	EXB L-1190	KF993675	MG597313
	14	*Pseudescherichia vulneris*	*P*	EXB L-1211	JQ958880	MG597314

DW3	15	*Bacillus circulans*	*F*	EXB L-1279	KM349203	MG597315
	16	*Micrococcus luteus*	*A*	EXB L-1261	KJ733861	MG597316
	17	*Microbacterium hydrocarbonoxydans*	*A*	EXB L-1250	JQ954857	MG597317
	18	*Exiguobacterium aestuarii*	*F*	EXB L-1244	FJ462716	MG597318
	19	*Exiguobacterium arabatum*	*F*	EXB L-1278	JF775422	MG597319
	20	*Exiguobacterium panipatensis*	*F*	EXB L-1260	EF519705	MG597320
	21	*Exiguobacterium profundum*	*F*	EXB L-1270	KM873375	MG597321

DW4	22	*Acinetobacter junii*	*P*	EXB-L-1308	EU862296	MG597322
	23	*Haematomicrobium sanguinis*	*A*	EXB-L-1326	EU086805	MG597323
	24	*Bacillus cereus*	*F*	EXB-L-1176	GU568201	MG597324
	25	*Brevibacterium casei*	*F*	EXB-L-1336	HM012705	MG597325
	26	*Exiguobacterium panipatensis*	*F*	EXB-L-1316	EF519705	MG597326
	27	*Exiguobacterium aestuarii*	*F*	EXB-L-1327	FJ462716	MG597327
	28	*Staphylococcus saprophyticus*	*F*	EXB-L-1314	AB697718	MG597328


*DW1, dishwasher 1; DW2, dishwasher 2; DW3, dishwasher 3; DW4, dishwasher 4; ^∗^, Phyla; P, Proteobacteria, F, Firmicutes, A, Actinobacteria. Strain ID represents the isolate identification after deposition (as ‘EXF’ for fungal and ‘EXB-L’ for bacterial isolates) at the Microbial Culture Collection Ex (*MRICUL EX*).*

### Growth Media and Conditions

To determine the optimal growth conditions and to evaluate the biofilm-forming capabilities of microorganisms obtained in this study, we selected 7 bacterial isolates from each of the four dishwashers providing a total of 28 bacterial isolates (**Table 2**). Selections of isolate were made between different phylogenetically diverse bacterial species in each dishwasher. These isolates were subcultured from frozen glycerol stocks onto LB (Luria-Bertani) agar plates and incubated for 24 h at 37°C. A single colony of each bacterial isolate was inoculated into 5 ml LB media tubes, incubated overnight at 37°C while shaken at 200 rpm.

### *In Vitro* Bacterial Multispecies Biofilm Cultivation

The seven selected isolates from each dishwasher (**Table 2**) were screened for biofilm formation as single species and in four-species combinations as described previously (Ren et al., 2015; Røder et al., 2015) with few modifications. Serial 10-fold dilutions of bacterial cultures were performed from overnight grown cultures (in LB media) where 1 ml of the dilutions were inoculated with 29 ml fresh LB media, incubated overnight at 37°C and shaking at 200 rpm. Cell cultures in exponential phase (OD_600_ between 0.3 and 0.7) were then selected, centrifuged at 8000 rpm (10 min, 21°C), washed with 1x phosphate buffer saline (PBS) and re-suspended in 10% w/v LB media (reduced). The optical density OD_600_ of each bacterial culture was then adjusted to 0.15 in the reduced LB media. Biofilm cultivation assay was performed using 96-well microtiter plates (NUNC, Roskilde, Denmark) and peg lids (NUNC-TSP lid system, Roskilde, Denmark) placed on top of the plates, also referred to as the Calgary method (Ceri et al., 1999). A total of 150 μl as mono-species or four mixed species (37.5 μl of each species) cultures were added to each well. Each plate contained the representative mono-species cultures. 150 μl 10% LB served as blank. Plates were incubated at 25°C for 24 h.

### Network Analysis Data

While competing for same resources, bacteria present in the same environment potentially co-occur or exclude each other (Roggenbuck et al., 2014). This relationship was characterized by generating the Spearman co-occurrence network (Barberán et al., 2012). The four selected dishwasher in this study, sequenced using Illumina MiSeq platform and taxonomic classifications of the 16S rRNA gene sequences based on RDP classifier, were described previously (Raghupathi et al., 2018). Sequence raw reads (SRR) (**Table 1**) from these dishwashers were made available to NCBI Sequence Read Archive (SRA) under the Bioproject ID: PRJNA315977. The network and predicted interactions were generated on the basis of relative counts of different bacterial genera that had more than 50 sequence observations and represented in 50% of the samples (*n* > 2, *N* = 4). We present correlation data for log transformed counts using CoNet 1.0b6 plugin in Cytoscape 3.2.1. The correlations were made on the basis of in-built non-parametric Spearman correlation coefficient with a minimal cut-off threshold of *r* ≥|0.85| (*p* << 0.01, Bonferroni corrected).

### *In Vitro* Cultivation of Bacterial–Fungal Biofilms

The bacterial isolates from DW4 were prepared as mentioned above. The fungal strain *E. dermatitidis* genotype A (EXF-9777), also isolated from DW4 (**Table 2**), was subcultured from frozen glycerol stocks onto MEA, supplemented with 0.05 g/l chloramphenicol and incubated 3–5 days at 37°C. A single colony of the black yeast was then inoculated into 5 ml 10% LB media tubes and incubated at 37°C while shaken at 200 rpm until an OD_600_ of approximately 0.7 was reached. Then, with the aim to work with a uniform culture media which will provide a common niche for both bacteria and fungi, LB media was replaced with 10% LB and OD_600_ adjusted to 0.15. A total of 150 μl as mono-species (bacteria/fungi) cultures or 30 μl for each species in five mixed species (four bacteria and *E. dermatitidis*) combinations were added to each well. Also, each plate contained the representative 75 μl of mono-species bacterial cultures together with 75 μl fungal cultures. Plates were incubated at 25°C for 24, 48, and 96 h. 150 μl 10% LB served as blank.

### Biofilm Quantification and Screening for Synergistic Interactions

Mixed species and monospecies biofilm cultivation in a 96-well Calgary Biofilm Device (CBD) and its quantification using 1% w/v crystal violet were performed as described previously (Ren et al., 2015; Røder et al., 2015). We classified synergy, as and when the measured absorbance from the CBD assay of the multispecies biofilm (MSB) being greater than that of the best single strain (BSS) biofilm producer present in the relevant combination when taking standard errors into account, i.e., (Abs_590_ MSB - Standard error) > (Abs_590_ BSS + Standard error) = Synergy, while (Abs_590_ MSB + Standard error) < (Abs_590_ BSS - Standard error) = No synergy (Ren et al., 2015). In case of bacterial–fungal biofilms, synergy was when the absorbance of multispecies bacterial–fungal biofilm was greater than that of the best single strain biofilm producer present together with the fungi (BSS) in the relevant combination when taking standard errors into account. Fold change (*F_d_*) is represented as ratio of the biofilm biomass of multispecies consortia with/without fungi to its best biofilm producer with/without fungi within the respective consortia, i.e., Fold change = Abs_590_ MSB - Standard error/Abs_590_ (BSS + Standard error). Hence, consortia with an *F_d_* > 1 are designated as synergistic. The above cultivation and quantification of biofilm was performed with three technical replicates and the assay was performed at three different times.

### *In Vitro* Establishment of Multispecies Biofilm on Dishwasher Rubber and Plastic Material and Its Quantification

Two four-species bacterial consortia from DW4 that showed an overall increase in biofilm formation in all trials, were tested for the incorporation of *E. dermatitidis* using a 24 well plate; as this fungus was found to be present on DW4 rubber seal. Enumeration of fungal and bacterial cells from the biofilm formed on wells was done using fluorescent associated cell sorting system BD FACS Calibur (BD Biosciences). The biofilm on the bottom of the plates were washed gently and the attached cells were scrapped-off, homogenized in 500 μl 1X PBS and transferred into micro-centrifuge tubes. The fungal cells were selectively stained using Calcofluor White Stain (Sigma–Aldrich) to differentiate from bacterial cells.

Further, the biofilm formation on three different types of elastomer; EPDM [ethylene propylene diene monomer (M-class)] referred to as 17, 18, 19 and three different types of polypropylene (PP) (C_3_H_6_)*_n_* referred to as 1, 2, 3; used in dishwasher industry were tested. The elastomer and plastic material were cut into slices of 1 cm^2^ size (with active surface 2 cm × 1 cm) and sterilized by autoclaving at 121°C for 15 min. Bacterial and fungal cultures were prepared as described above. 24-well cell culture plates (TPP^®^ cat. no. 92024, Sigma–Aldrich, United States) were used to cultivate the biofilms on artificial materials of EPDM and PP. A total of 1250 μl for monospecies bacterial or fungal cultures or four mixed species (312, 5 μl of each bacterial culture), or five mixed species (250 μl of each bacteria and fungi cultures) combinations were added to each well. The same volume of 10% LB medium was added as blank. After inoculation, sterile elastomer or plastic parts were aseptically added into the plates. The plates were incubated at 25°C for 24, 48, and 120 h. The biofilm assays were performed three times on different days with three technical replicates each time.

The crystal violet method was applied to quantify biofilms formed on EPDM/PP (Ren et al., 2015; Røder et al., 2015) as follows. Briefly, after incubation, in order to wash off loosely attached cells and planktonic fractions, the EPDM/PP substrates were transferred using sterile forceps successively to three 24-well microtiter plates containing 1200 μl of 1X PBS buffer per well, followed by staining of the biofilms formed on the EPDM/PP with 1250 μl of an aqueous 1% (w/v) CV solution. After 20 min, the EPDM/PP substrate was rinsed three times with 1X PBS and de-stained in 1250 μl 96% ethanol in each well of a new plate. After 20 min, the absorbance was measured as described above.

## Results

### Variation in Total Cultivation Community Structure across Four Different Dishwashers

Among the dishwashers that were screened for viable microbial population within 1 cm^2^ isolation area from four DW, a total of 80 isolates (64 bacterial and 16 fungal) were obtained (Supplementary Table S1). Isolates from DW1 contained seven different fungal species and 20 different bacterial species. The fungal isolates belonged to four different classes viz. Saccharomycetes, Chaetothyriomycetes, Sordariomycetes, and Urediniomycetes. Majority of the isolated bacterial species belonged to *Proteobacteria*; and others belonged to four different bacterial phyla. DW2 had 3 fungal species and 18 different bacterial species belonging to 3 different bacterial phyla. Ten Gram-positive isolates belonging to two bacterial phyla, *Firmicutes* and *Actinobacteria* and no fungal isolates were obtained from DW3. Majority of these bacterial isolates belonged to the genus *Exiguobacterium*. Isolates from DW4 contained three different fungal species belonging to 3 fungal classes and 16 different bacterial species. The 16 isolates belonged to 4 different bacterial phyla. Bacterial isolates from DW3 and DW2 were represented by two or three families (DW3: *Microbacteriaceae* and *Bacillaceae*; DW2:-*Enterobacteriaceae*, *Micrococcaceae*, and *Moraxellaceae*) respectively. Bacterial isolates from DW1 and DW4 were represented by five families (DW1: *Pseudomonadaceae*, *Brucellaceae*, *Enterobacteriaceae*, *Xanthomonadaceae*, and *Bacillaceae*; DW4: *Moraxellaceae*, *Bacillaceae*, *Staphylococcaceae*, *Brevibacteriaceae*, and *Micrococcaceae*). DW2 and DW4 contained the black yeast *E. dermatitidis*, represented by two different genotypes, of which, the clinically relevant genotype A was present in both DWs. Previous results showed the most abundant microbial taxa in these four DW samples identified by 16S rRNA and ITS gene marker based amplicon sequencing (Raghupathi et al., 2018). Most abundant bacterial taxa belonged to genera like *Exiguobacterium, Gordonia, Nesterenkonia, Ochrobactrum, Chryseobacterium, Stenotrophomonas, Pseudomona*, and *Acinetobacter*. Most abundant fungal taxa in these four DW samples were represented by genera *Candid*a, *Cryptococcus, Rhodotorula*, and *Exophiala* (Raghupathi et al., 2018; Supplementary Figure S1).

**FIGURE 1:**
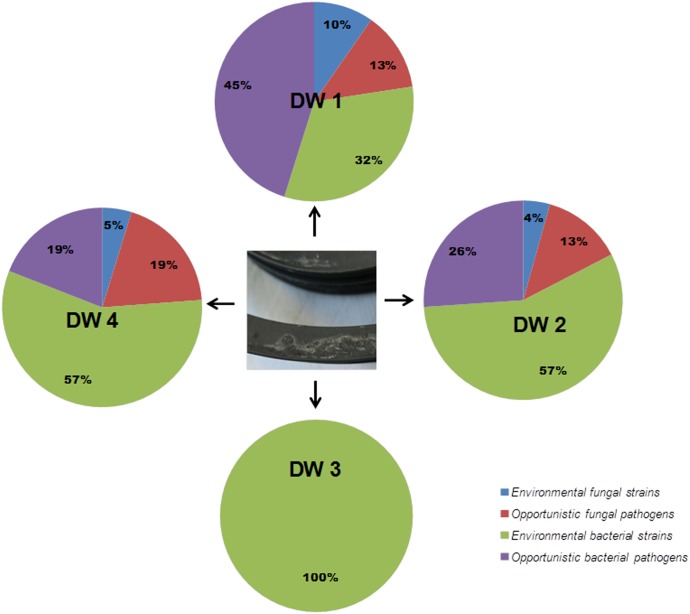
Distribution of microbial population isolated from the rubber seals of 4 dishwashers (DWs). After isolation and identification of both bacteria and fungi, isolates were classified as environmental or opportunistic pathogenic strains based on the taxonomic literature (de Hoog et al., 2014; BMSAB, 2017) in each dishwasher; DW1, dishwasher 1; DW2, dishwasher 2; DW3, dishwasher 3; DW4, dishwasher 4.

Bacteria classified as opportunistic pathogens like *Pseudomonas aeruginosa*, *Ochrobactrum pseudintermedium*, *Klebsiella oxytoca*, and *Acinetobacter junii* and opportunistic fungal pathogens like *E. dermatitidis*, *Candida parapsilosis*, *Rhodotorula mucilaginosa*, and *Fusarium oxysporum* species complex (FOSC) were isolated from these dishwashers. Bacterial and fungal isolates from DW1, 2, and 4 were represented by various opportunistic pathogens whereas; the isolates from DW3 were represented by non-pathogenic “environmental” strains (**Figure 1**). These classifications were made based on known fungal and bacterial taxonomic literatures [de Hoog et al., 2014; Whitman WB, 11th ed. Bergey’s Manual of Systematics of Archaea and Bacteria (BMSAB, 2017)].

### Multi-Species Interactions Enhance Biofilm Biomass

**FIGURE 2:**
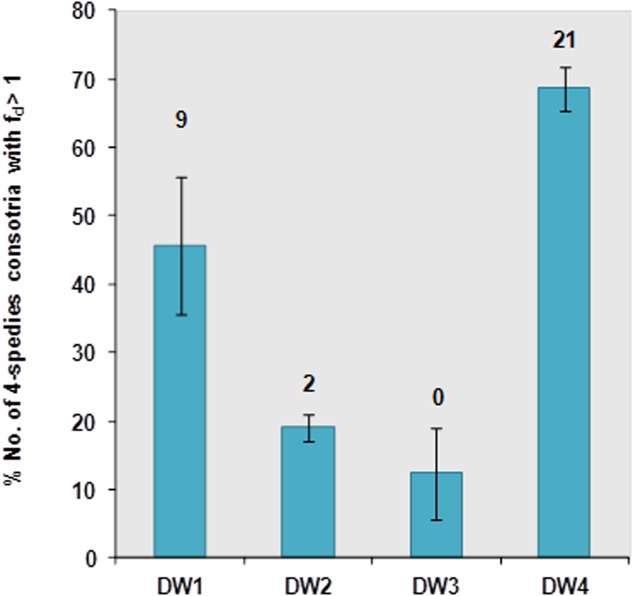
Percentage of synergistic (*f_d_*-value >1) four-species bacterial combinations (total; *N* = 35) from DW1-4 based on CV quantification after 24 h incubation at 25°C in 10% LB media. The number above each column bar indicates the total number of four species consortia having *f_d_*-value >1 in all replicates and trials. The experiment was performed at three different times with three technical replicates each time. The error bars denote the percentage mean ± standard error (*SE*) from three biological trails.

Screening for biofilm formation revealed that DW1 and DW4 had higher percentage of four-species consortia with *f_d_* > 1, thus considered to be synergistic in biofilm formation, compared to DW2 and DW3 (**Figure 2**). Overall 35 four-species combinations were tested per each DW, 140 combinations in total per experiment. Results showed that DW1, DW2, and DW4 had 9, 2, and 21 stable four-species combinations, respectively, [consistently synergistic (fold-change, *f_d_* > 1)] in all three trials. DW3 had no four-species combinations interacting synergistically across all trials. The absorbance measurements of single and four-species combinations and their corresponding fold-change (*f_d_*) calculated across the three biological trails are shown (Supplementary Table S2).

**FIGURE 3:**
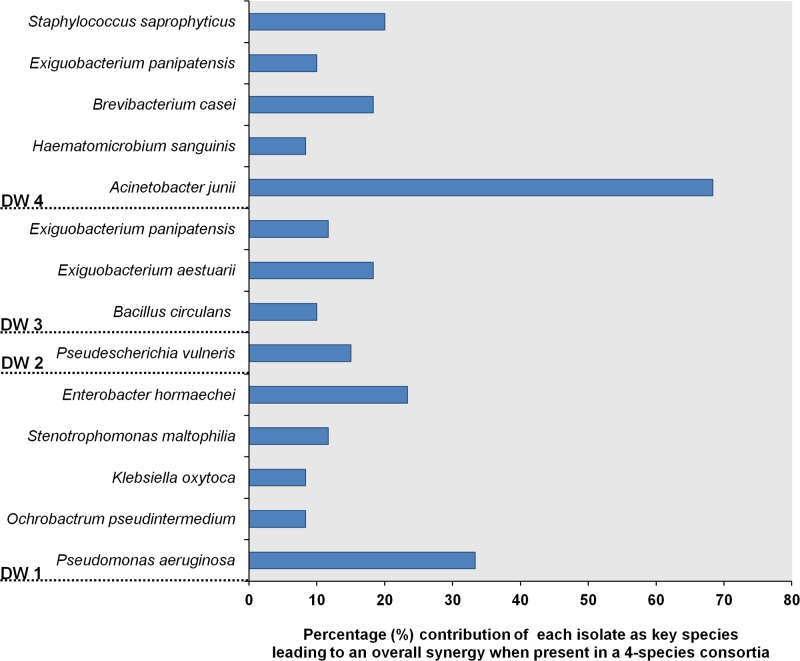
Isolates from dishwashers that were assessed as key species contributing to biofilm synergy when present in a four-species combination. The number of times an isolate contributing to synergy within a four-species consortium were summarized from three trials. Each isolate participated in 20 four species combinations/trial, thus *N* = 60 observations.

The four-species consortia were analyzed to identify the different species contributing as key biofilm producers when present within the given consortia. Therefore, the isolates that contributed more frequently to synergy in each four-species combination were obtained. The analysis performed across three trials gave a maximum count of 60 combinations per isolate (**Figure 3**). In DW1, four-species combinations containing *P. aeruginosa* and *Enterobacter hormaechei* were more likely to interact synergistically. In DW4, *Acinetobacter junii* was the most frequent isolate contributing to synergistic interactions. In DW2 and DW3, the frequency of each isolate to engage in a synergistic four-species biofilm varied among different bacterial members. *Escherichia vulneris* and *Exiguobacterium aestuarii* in different four-species combinations were more likely to interact synergistically in DW2 and DW3, respectively.

### Potential Interactions between Different Bacterial Taxa Using a Network Based Approach

**FIGURE 4:**
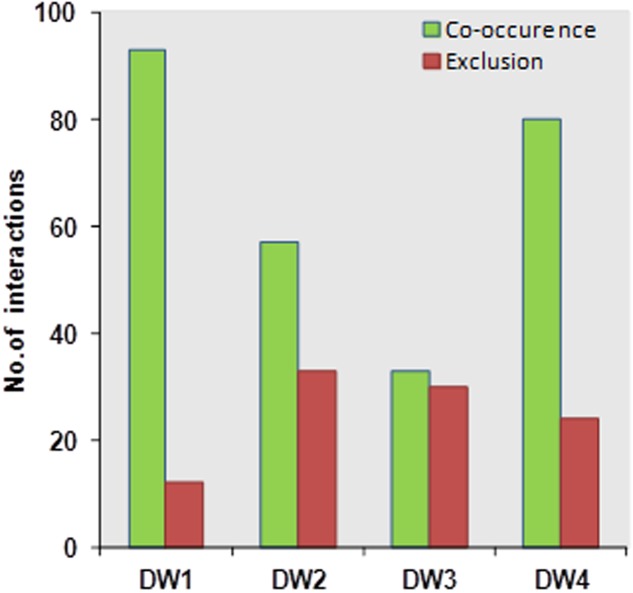
Network based analysis showing the number of co-occurrences and mutual exclusion interactions among bacterial genera identified in the four dishwasher systems generated based on Spearman correlation analysis.

Bacterial diversity based on 16S rRNA gene sequencing of these four DW biofilm communities was revealed in a previous study (Raghupathi et al., 2018). Significant pairwise interactions (*p* < 0.01) between different bacterial genera from these four DW samples were analyzed. The type of interaction, i.e., positive correlation hypothetically indicates symbiosis, mutualism or commensalism and negative correlation hypothetically indicates mutual exclusions, competition or parasitism (Roggenbuck et al., 2014). It was found that in DW1 and DW4, the numbers of positive correlations were higher than in DW2 and DW3 (**Figure 4**). The interaction networks within different bacterial genera identified in this study are presented (Supplementary Figure S2). The genera *Pseudomonas* and *Acinetobacter* had highest numbers of positive correlations suggesting a potential to co-exist with other bacterial genera.

### Bacterial–Fungal Biofilm Development

**FIGURE 5:**
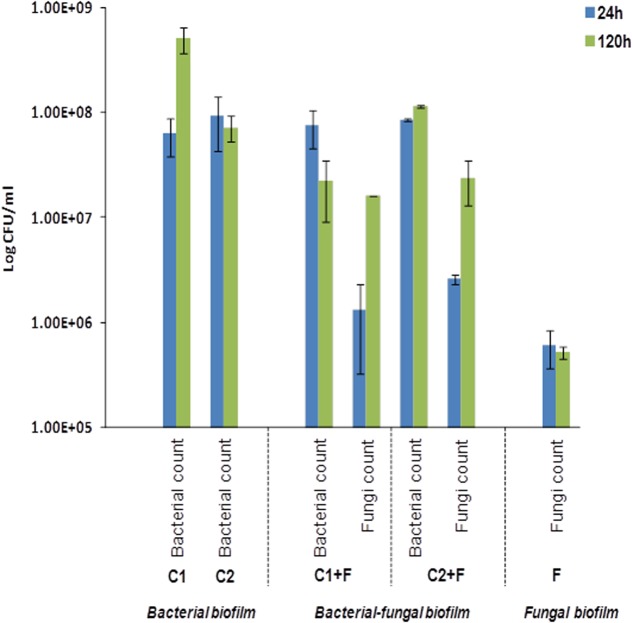
Establishment of fungal cells into bacterial consortia. Log_10_ CFU counts obtained after 24 and 120 h of incubation at 25°C harvested from biofilms formed on the wells using flow cytometry. Consortium C1 were composed of *A. junii*, *B. cereus*, *H. sanguinis*, and *E. aestuarii* species and consortium C2 were composed of *A. junii*, *B. cereus*, *B. casei*, and *E. aestuarii* species. C1 and C2 denote the total CFU counts from biofilm formed by the two four-species bacterial consortia with no fungal addition and not of individual isolates within the consortia. ‘C1+F’ and ‘C2+F’ denote the counts of total bacterial and *E. dermatitidis* cells when these consortia were co-cultured with the fungi *E. dermatitidis*. ‘F’ denotes the total cell counts of *E. dermatitidis* when present to form monospecies fungal biofilm. The error bars denote the mean cell counts ± *SE* from three biological trials.

*Exophiala dermatitidis* is known for its dominant presence in household DWs (Zalar et al., 2011; Zupančič et al., 2016). Therefore, its establishment within bacterial biofilms was investigated. Different four-species bacterial consortia from DW4 were tested for their ability to incorporate *E. dermatitidis* (see Supplementary Table S3). We found that two four-species bacterial consortia increased in its overall biofilm production when *E. dermatitidis* was included. One bacterial consortium (Consortium 1) was composed of *Acinetobacter junii* (EXB-L-1308), *Haematomicrobium sanguinis* (EXB-L-1326), *Bacillus cereus* (EXB-L-1176) and *Exiguobacterium aestuarii* (EXB-L-1327). The other bacterial consortium (Consortium2) was composed of *Acinetobacter junii* (EXB-L-1308), *Bacillus cereus* (EXB-L-1176), *Brevibacterium casei* (EXB-L-1336), and *Exiguobacterium aestuarii* (EXB-L-1327). It should be noted that the bacterial consortium 1 when present alone increased in cell numbers over time, however, the bacterial cell numbers reduced overtime in Consortium 1 in the presence of fungal cells. Consortium 2 showed no change in bacterial numbers and fungal numbers increased overtime. These results indicate a shift in population dynamics that could be observed due to resource competition and complex interactions between different microbial species. Interestingly, *E. dermatitidis* did not form biofilm when grown as fungal monocultures as it did not attach well to the surface of the Calgary biofilm device (CBD) indicated by its low cell numbers. However, when *E. dermatitidis* was introduced to multispecies bacterial biofilm, the cell numbers increased leading to the formation of the trans-kingdom biofilm (**Figure 5**).

### Industrial Implications

**FIGURE 6:**
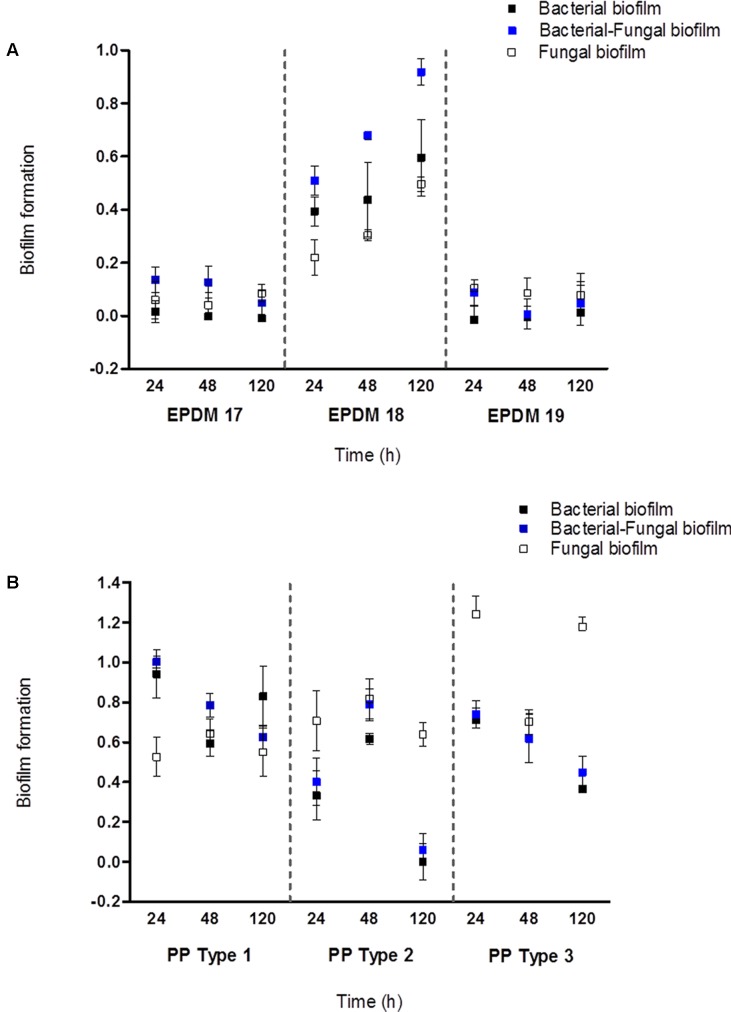
Microbial biofilm formation on EPDM and PP materials. **(A)** Biofilm establishment on three EPDM rubber types after 24, 48, and 120 h of incubation at 25°C. **(B)** Biofilm establishment on three PP types after 24, 48, and 120 h of incubation at 25°C. The biofilm establishement were absorbance (OD_590_) measurements quantified by 1% CV staining. The error bars denote the mean biofilm formation ± *SE* from three biological trails.

Synthetic surfaces in many machines and equipment, including household appliances and medical utensils, may become established with microbial biofilm overtime. This could contribute to risks associated with cross-contamination. As an applied aspect of this study, we wanted to assess the establishment and colonization of bacterial–fungal biofilms on different elastomer (EPDM) and polypropylene (PP) surfaces using bacterial Consortium 1 together with *E. dermatitidis*. This multispecies bacterial–fungal biofilm was best formed on elastomer 18, which constitute as the actual rubber material currently used in the industry for rubber seals. Biofilms were less successfully established on elastomer types 17 and 19 (**Figure 6A**). *E. dermatitidis* grown as a mono-species fungal biofilm also showed an increased attachment to elastomer 18 compared to elastomers 17 and 19 (**Figure 6A**). Thus, elastomer 18 represents a preferred surface for microbial biofilm formation. However, based on the absorbance measurements from microbial biomass formed on different PP surfaces; our observation point to PP surfaces providing an even better surface for microbial attachment (**Figure 6B**) than elastomers. Similar results were observed on bacterial–fungal biofilms using bacterial isolates in Consortia 2 (Supplementary Figure S3).

## Discussion

Survival of microorganisms in extreme environments is often associated with formation of complex biofilms attached on a suitable surface (Davey and O’toole, 2000). In domestic environments, biofilms were examined in tap water supply systems (Mullis and Falkinham, 2013; Burkowska-But et al., 2015; Lührig et al., 2015; Babič et al., 2016; Iakhiaeva et al., 2016; Ling et al., 2016) and in wet niches such as shower heads (Abe et al., 2016). In this study, we focussed on the isolation of microorganisms from biofilms formed on rubber seals of four dishwashers (DWs) and used these isolates to determine their biofilm forming abilities *in vitro*. Bacterial communities that colonized the rubber seals of DWs comprised a wide variety of environmental bacterial species together with a number of species represented as opportunistic pathogens.

The composition of the microbial communities differed considerably among the four DWs. Microbial species obtained from these samples were well represented based on their abundance levels at their genus level (Raghupathi et al., 2018). These results show that most abundant microbial representatives identified by sequencing approach, remained viable in these extreme systems. The dominant bacterial genus, *Exiguobacterium* was isolated in three out of four DWs. Different species of this genus were known for their ability to proliferate in extreme natural environments like hot, alkaline and marine environments (Vishnivetskaya et al., 2009). Another highly represented bacterial genus was *Bacillus. Bacillus* is ubiquitous in domestic environments (Gorny et al., 1999; Park et al., 2006) and is widely used in industry as a microbial indicator for cleaning procedures during washing cycles (Ståhl Wernersson et al., 2004; Lee et al., 2007; Nicolella et al., 2011). The diversity of fungi isolated from DWs was in accordance with previous studies (Zalar et al., 2011; Döğen et al., 2013; Gümral et al., 2016; Zupančič et al., 2016). Black yeasts, *E. dermatitidis* and *E. phaeomuriformis* were represented in most dishwashers; followed by white yeasts, *Candida parapsilosis* and red yeasts, *Rhodotorula mucilaginosa*. These four fungal species were classified as opportunistic human pathogens (Hiruma et al., 1993; Lunardi et al., 2006; De Almeida et al., 2008; Sudhadham et al., 2008; Trofa et al., 2008; Russo et al., 2010; Kondori et al., 2011; Silva et al., 2012) and with their presence in household DWs, they could represent a potential source for indoor infections (Zupančič et al., 2016).

Bacterial interactions play a major role in shaping and maintaining the diversity within bacterial communities (HilleRisLambers et al., 2012) and also influence the balance between cooperating and competing phenotypes (Nadell et al., 2016). Studies have elucidated the coexistence patterns among microbial groups from a variety of ecosystems using microbial correlation networks (Eiler et al., 2012; Kittelmann et al., 2013; Zhalnina et al., 2013). However, little is known on whether these coexistence patterns reflect the actual biogenic relationships and interactions *in situ*. In this study, we analyzed the co-occurrence patterns between the different bacterial taxa in the four DW samples. Positive and negative correlations of bacterial taxa were accounted to the genus level. DW1 and DW4 had higher number of positive correlations compared to DW2 and DW3. Also, when screened for synergistic multispecies biofilm, it was found that DW1 and DW4 had higher numbers of four-species combinations interacting synergistically leading to an overall increase in biomass.

Biofilm levels of the four-species consortia when further examined and compared to the levels of biofilm production of each isolate under monospecies conditions, it was revealed that *P. aeruginosa* and *A. junii*, isolated from DW1 and DW4, respectively, were found to contribute as best biofilm producers that included poor or non-biofilm producing isolates, increasing the overall biofilm formation within the included consortia. Likewise, co-association networks revealed that the genera *Pseudomonas* and *Acinetobacter* had higher number of positive correlations suggesting a potential to cooperate with other bacterial genera. These observations could support our previous evidence where DW1 and DW4 had higher percentage of synergistically interacting four-species biofilm. Likewise, the co-association networks revealed that the genera *Exiguobacterium* and *Micrococcus* had higher numbers of negative correlations signaling competition or exclusion to other bacteria. In DW2 and DW3, most combinations included isolates belonging to the genera *Exiguobacterium* and *Micrococcus* and the observed number of synergistic four-species consortia were much lower than what was seen in DW1 and DW4. Interestingly, these findings demonstrate an observed trend between the correlation detection technique and *in vitro* multispecies biofilm assessments, where coexistence of bacterial members within these ecological systems could contribute to multispecies biofilm formation.

Synergy impacts bacterial composition in multispecies biofilms and their overall biomass (Burmølle et al., 2014). Such multispecies biofilms are tolerant against antimicrobials compared to their monospecies equivalents (Schwering et al., 2013; Lee et al., 2014). We have characterized the interactions within different bacterial species and how they impact each other during biofilm development, both under mono and mixed species cultures. Later, the ability of selected mixed bacterial consortia to incorporate the polyextremophile *E. dermatitidis*, (Zalar et al., 2011; Poyntner et al., 2016) the prevalent fungal species in DW systems, was assessed. Fungi and bacteria play important roles in promoting the survival of their interacting partners (Frey-Klett et al., 2011). Such complex biofilms can be beneficial to all microbial partners, but can be detrimental to the human host (Minerdi et al., 2008; Ghannoum, 2016; Kalan et al., 2016). Our results show that when bacterial consortia were supplemented with *E. dermatitidis*, the biomass production and the numbers of bacteria were stimulated together with the growth of fungal partner in the mixed biofilm. The observations that the bacterial community of DWs facilitating the growth of an opportunistic pathogenic fungus and mixed bacterial–fungal biofilm established on commonly used industrial surfaces (EPDM 18 and PP) complementing their persistence and growth; represent significant findings with scientific and applied implications. Though these observations are similar to the results obtained in other studies investigating mixed species biofilms like *Candida albicans*, an opportunistic pathogenic fungi (Seneviratne et al., 2007; Harriott and Noverr, 2011; Pammi et al., 2013), it should be noted that the studies were made using one fungi and single bacteria in co-cultures; whereas, in this study, we present the establishment of an opportunistic black yeast pathogen into mixed bacterial consortium comprising of four species. Further, the formation of bacterial and fungal biofilms on dishwasher related environments emphasize the importance of interactions played between different microbial species and their change in population dynamics across kingdoms during biofilm development.

In summary, our main findings include the existence of synergistic interactions observed during biofilm formation between bacteria isolated from different DWs where, *A. junii* and *P. aeruginosa* were recognized as the best biofilm producers and important contributors to synergy. This finding corresponds with network based co-occurrence analysis where these two bacterial genera in dishwasher systems, account to most positive correlations observed. In addition, mixed bacterial biofilms could incorporate the opportunistic yeast pathogen, *E. dermatitidis* and facilitate its establishment on rubber seals and other surfaces. The enhancement of trans-kingdom biofilm formation on rubber surface used in DWs suggests that microbes surviving these environments have been selected by their ability to engage in synergistic biofilm formation. With our study, we have shown that our experimental model has the capacity to reveal new and unique features of these complex and dynamic microbial communities. Additionally, our observations and methodology could have important implications for future design and maintenance of house-hold and medical appliances, as these systems could present as a source of domestic cross-contamination and human infections.

## Ethics Statement

In this study, field sampling was performed, and to our knowledge, no endangered or protected species were involved. All of the samples studied here were obtained from the discussed sampling areas, for which permission was obtained from the owners.

## Author Contributions

SS, NG-C, and MB designed the study. JZ and PR performed the experiments and analyzed the data. JZ, PR, KH, NG-C, MB, and SS compiled the manuscript.

## Conflict of Interest Statement

The authors declare that the research was conducted in the absence of any commercial or financial relationships that could be construed as a potential conflict of interest.

## Footnotes

1 http://rdp.cme.msu.edu
