# Supplementary material for: Synergistic Interactions in Microbial Biofilms Facilitate the Establishment of Opportunistic Pathogenic Fungi in Household Dishwashers
Source: Front Microbiol. 2018 Jan 30;9:21. doi: 10.3389/fmicb.2018.00021 (PMC5797641; doi:10.3389/fmicb.2018.00021)
Supplement: Supplementary file 1 [file Data_Sheet_1.PDF]

Supplementary information

Table S1: Bacterial and fungal isolates obtained as pure cultures from DW associated biofilm formed on 1 cm^2 rubber seals. 16S rRNA 16S ribosomal RNA gene; **LSU** 26S ribosomal RNA gene; **ITS** internal transcribed spacer 1, 5.8S ribosomal RNA gene, and internal transcribed spacer 2; ***tef***, translation elongation factor 1-alpha (EF1a) gene; **FOSC** *Fusarium oxysporum* species complex. Strain ID represents the isolate identification after deposition (as 'EXF' for fungal and 'EXB-L' for bacterial isolates) at the Microbial Culture Collection Ex (MRICUL EX).

Closest Relative	Phylum	DNA based identification method	Strain ID EXF- / EXB L-	Accession number of the closest relative
**DW1**				
Bacillus cereus	Firmicutes	16S rRNA	EXB L-1175	KC969074
Bacillus pumilus	Firmicutes	16S rRNA	EXB L-1225	AJ494726
Bacillus sp.	Firmicutes	16S rRNA	EXB L-1177	HM989921
Bacillus thuringiensis	Firmicutes	16S rRNA	EXB L-1173	JX035937
Brachybacterium paraconglomeratum	Actinobacteria	16S rRNA	EXB L-1160	FJ172038
Chryseobacterium sp.	Bacteroidetes	16S rRNA	EXB L-1165	JN545042
Comamonas testosteroni	Proteobacteria	16S rRNA	EXB L-1141	AY247415
Enterobacter cancerogenus	Proteobacteria	16S rRNA	EXB L-1132	FJ009375
Enterobacter hormaechei	Proteobacteria	16S rRNA	EXB L-1135	KP303395
Enterobacter sp.	Proteobacteria	16S rRNA	EXB L-1129	KM979225
Klebsiella oxytoca	Proteobacteria	16S rRNA	EXB L-1137	CP011636
Kurthia gibsonii	Firmicutes	16S rRNA	EXB L-1146	AB271738
Leucobacter sp.	Actinobacteria	16S rRNA	EXB L-1152	KC550185
Lysinibacillus fusiformis	Firmicutes	16S rRNA	EXB L-1140	DQ333300
Ochrobactrum pseudintermedium	Proteobacteria	16S rRNA	EXB L-1130	KF026284
Pseudomonas aeruginosa	Proteobacteria	16S rRNA	EXB L-1125	KR911837
Pseudomonas alcaligenes	Proteobacteria	16S rRNA	EXB L-1113	AF390747
Pseudomonas putida	Proteobacteria	16S rRNA	EXB L-1149	KJ735915
Sphingobacterium spiritivorum	Bacteroidetes	16S rRNA	EXB L-1227	EF090267
Stenotrophomonas maltophilia	Proteobacteria	16S rRNA	EXB L-1167	KP185140
Candida parapsilosis	Ascomycota	LSU	EXF-9745	KJ481229
Candida pararugosa	Ascomycota	LSU	EXF-9751	GU904205
Clavispora lusitaniae	Ascomycota	LSU	EXF-9744	KF728663
Exophiala phaeomuriformis genotype 1	Ascomycota	ITS	EXF-9735	KP034987
Fusarium oxysporum species complex	Ascomycota	*tef*	EXF-9737	KP761169
Meyerozyma guilliermondii	Ascomycota	LSU	EXF-9759	KJ481231
Rhodotorula mucilaginosa	Basidiomycota	LSU	EXF-9755	KC442283
**DW2**				
Acinetobacter lwoffii	Proteobacteria	16S rRNA	EXB L-1215	LN774665
Acinetobacter sp.	Proteobacteria	16S rRNA	EXB L-1191	AY486382
Aerococcus sp.	Firmicutes	16S rRNA	EXB L-1205	EU376006
Bacillus cereus	Firmicutes	16S rRNA	EXB L-1223	KP988025
Enterobacter sp.	Proteobacteria	16S rRNA	EXB L-1204	KM979225
Pseudescherichia vulneris	Proteobacteria	16S rRNA	EXB L-1211	JQ958880
Exiguobacterium aestuarii	Firmicutes	16S rRNA	EXB L-1196	FJ462716
Exiguobacterium panipatensis	Firmicutes	16S rRNA	EXB L-1201	EF519705
Exiguobacterium sp.	Firmicutes	16S rRNA	EXB L-1196	EU159578
Kocuria rhizophila	Actinobacteria	16S rRNA	EXB L-1199	AY030315
Kocuria salsicia	Actinobacteria	16S rRNA	EXB L-1221	GQ352404
Lactococcus lactis subsp. lactis	Firmicutes	16S rRNA	EXB L-1213	KR732324
Leclercia sp.	Proteobacteria	16S rRNA	EXB L-1203	JX949970
Micrococcus luteus	Actinobacteria	16S rRNA	EXB L-1190	KF993675
Micrococcus sp.	Actinobacteria	16S rRNA	EXB L-1212	EU379020
Pseudomonas psychrotolerans	Proteobacteria	16S rRNA	EXB L-1186	KM019821
Pseudomonas sp.	Proteobacteria	16S rRNA	EXB L-1220	AM945563

Rothia sp.	Actinobacteria	16S rRNA	EXB L-1189	EU135638
Candida parapsilosis	Ascomycota	LSU	EXF-9760	KJ481228
Exophiala dermatitidis genotype A	Ascomycota	ITS	EXF-9487	DQ826738
Exophiala dermatitidis genotype C	Ascomycota	ITS	EXF-9463	JF766671
Rhodotorula mucilaginosa	Basidiomycota	LSU	EXF-9756	KP087899
**DW3**				
Bacillus cereus	Firmicutes	16S rRNA	EXB L-1263	KC969074
Bacillus circulans	Firmicutes	16S rRNA	EXB L-1279	KM349203
Exiguobacterium aestuarii	Firmicutes	16S rRNA	EXB L-1244	FJ462716
Exiguobacterium arabatum	Firmicutes	16S rRNA	EXB L-1278	JF775422
Exiguobacterium panipatensis	Firmicutes	16S rRNA	EXB L-1260	EF519705
Exiguobacterium profundum	Firmicutes	16S rRNA	EXB L-1270	KM873375
Exiguobacterium sp.	Firmicutes	16S rRNA	EXB L-1269	EU159578
Microbacterium hydrocarbonoxydans	Actinobacteria	16S rRNA	EXB L-1250	JQ954857
Microbacterium sp.	Actinobacteria	16S rRNA	EXB L-1272	FR774577
Micrococcus luteus	Actinobacteria	16S rRNA	EXB L-1261	KJ733861
**DW4**				
Acinetobacter junii	Proteobacteria	16S rRNA	EXB-L-1308	EU862296
Acinetobacter sp.	Proteobacteria	16S rRNA	EXB-L-1324	EU705470
Haematomicrobium sanguinis	Actinobacteria	16S rRNA	EXB-L-1326	EU086805
Bacillus amyloliquefaciens	Firmicutes	16S rRNA	EXB-L-707	JX036499
Bacillus cereus	Firmicutes	16S rRNA	EXB-L-1176	GU568201
Bacillus horneckiae	Firmicutes	16S rRNA	EXB-L-1313	FR749913
Brachybacterium paraconglomeratum	Actinobacteria	16S rRNA	EXB-L-1311	FJ172038
Brevibacillus sp.	Firmicutes	16S rRNA	EXB-L-1330	GQ927158
Brevibacterium casei	Actinobacteria	16S rRNA	EXB-L-1336	HM012705
Brevibacterium sanguinis	Actinobacteria	16S rRNA	EXB-L-1305	AJ564859
Exiguobacterium aestuarii	Firmicutes	16S rRNA	EXB-L-1327	FJ462716
Exiguobacterium panipatensis	Firmicutes	16S rRNA	EXB-L-1316	EF519705
Exiguobacterium profundum	Firmicutes	16S rRNA	EXB-L-1335	KF269103
Exiguobacterium sp.	Firmicutes	16S rRNA	EXB-L-1331	EF519705
Microbacterium paraoxydans	Actinobacteria	16S rRNA	EXB-L-1310	DQ350825
Staphylococcus saprophyticus	Firmicutes	16S rRNA	EXB-L-1314	AB697718
Candida parapsilosis	Ascomycota	LSU	EXF-9764	EU056283
Exophiala dermatitidis genotype A	Ascomycota	ITS	EXF-9777	DQ826738
Exophiala dermatitidis genotype A2	Ascomycota	ITS	EXF-9778	FJ387565
Exophiala phaeomuriformis genotype 1	Ascomycota	ITS	EXF-9779	KP034987
Rhodotorula mucilaginosa	Basidiomycota	LSU	EXF-9762	AF335986

Figure S1: Heat map of most abundant (A) bacterial and (B) fungal genera identified by 16s rRNA and ITS gene based sequencing done in a previous study (Raghupathi *et al.*, 2017). The scaled heat maps were generated using log-transformed bacterial and fungal abundances and clustered based on 'coniss'. The heatmaps were created using various R packages: gplots, vegan, rioja and *Rcolorbrewer* available for *Rgui* 3.2.0.

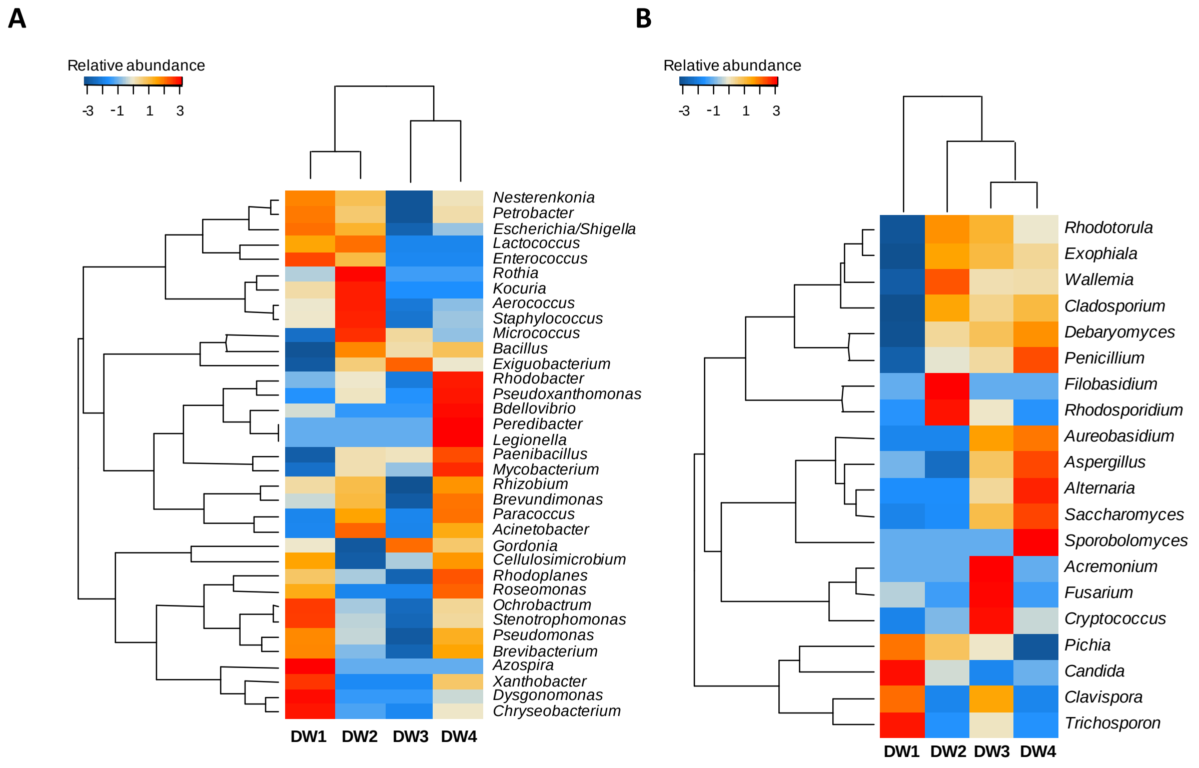

Figure S2: Bacterial co-occurrences by network analysis and its parameters. The type of interaction of one bacterial genus to the other bacterial genera from network based analysis and the network parameters. 'Green' connectors indicate 'positive correlations' signalling cooperation and 'red' connectors indicate 'negative correlations' signalling mutual exclusion. The significant networks ($p < 0.01$) were generated using log-transformed bacterial abundance and bacterial taxa that were present in sample ($n > 2$), classified to the genus level and represented in the isolate collection (Table 1) of dishwashers.

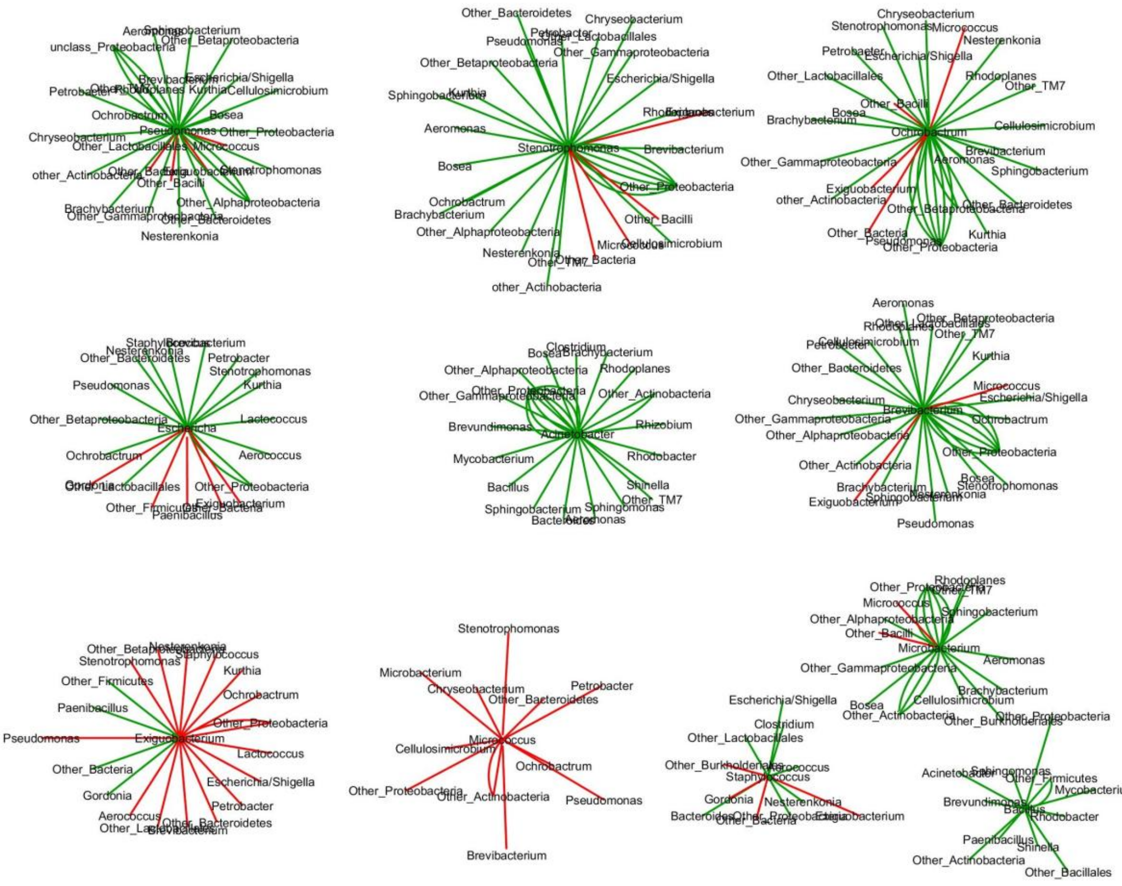

Clustering coefficient :	0.740	Number of nodes :	106
Connected components :	5	Network density :	0.121
Network diameter :	4	Network heterogeneity :	0.539
Network radius :	1	Isolated nodes :	0
Network centralization :	0.119	Number of self-loops :	0
Shortest paths :	3520 (31%)	Multi-edge node pairs :	0
Characteristic path length :	2.0375	Analysis time (sec) :	0.393
Avg. number of neighbors :	12.736		

Figure S3: Microbial biofilm formation of Consortia 2 on EPDM and PP materials A) Biofilm establishment on three EPDM rubber types and **B)** Biofilm establishment on three PP types after 24, 48 and 120 hours of incubation at 25 °C. The biofilm establishedment were absorbance (OD_{590}) measurements quantified by 1% CV staining. The error bars denote the mean ± S.E.M from three biological trails.

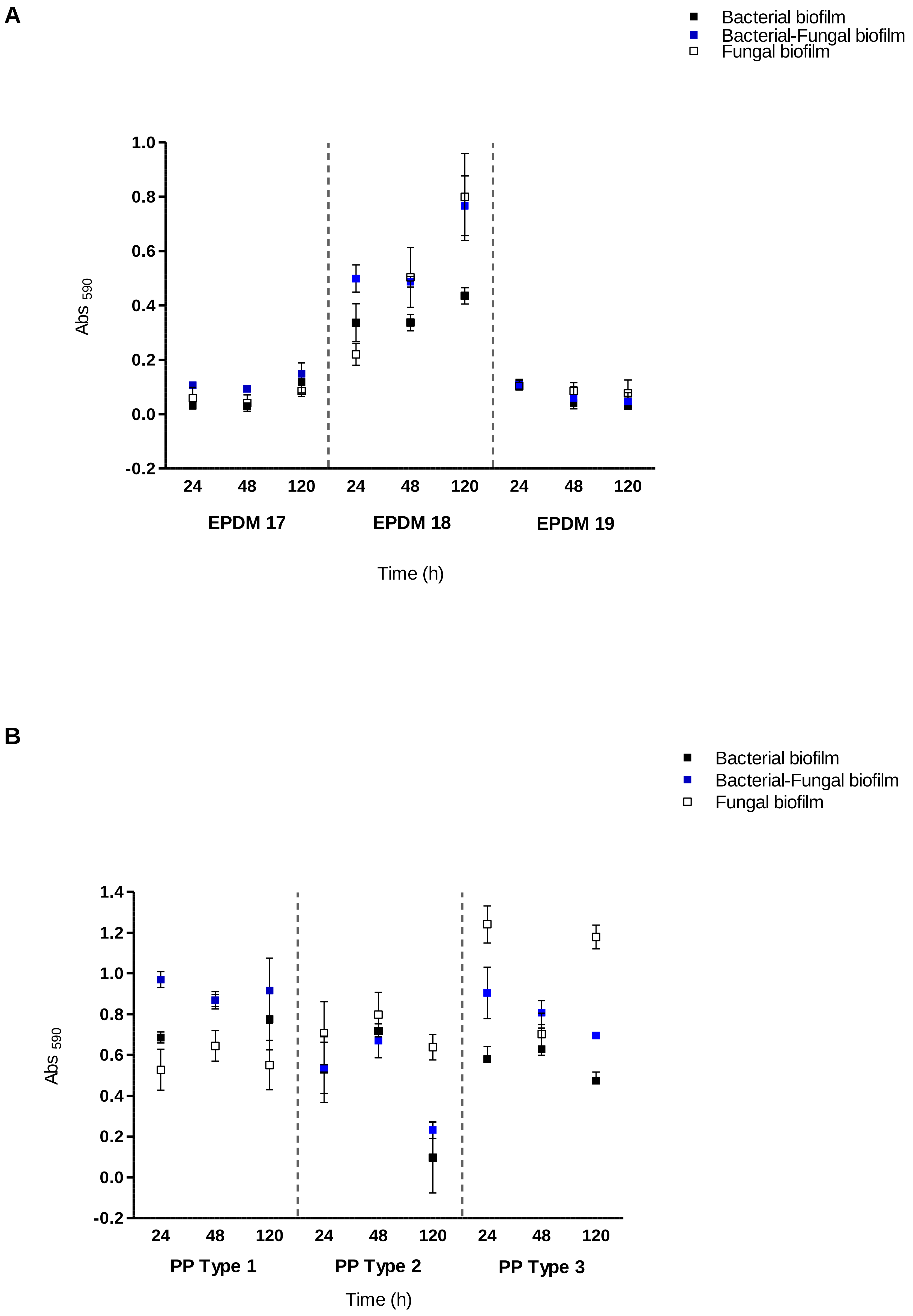

Table S2: Biofilm formed by single and combinations of 4-species observed in samples DW1, DW2, DW3 and DW4. Biofilm quantification in 10%LB was done after 24 hours of incubation at 25°C by crystal violet staining and absorbance measured at 590 nm. $\mathbf{F_d}$ is the ratio of (Abs 590 multispecies biofilm – Std Err) / (Abs 590 best single species+ Std Err) or (Abs 590 multispecies biofilm + Std Err) / (Abs 590 best single species - Std Err) calculated across the three biological trials

	Biofilm formation (Abs590)			Trial 1	Trial 2	Trial 3		Biofilm formation (Abs590)			Trial 1	Trial 2	Trial 3
Isolate	Trial 1	Trial 2	Trial 3	F_d	F_d	F_d	Isolate	Trial 1	Trial 2	Trial 3	F_d	F_d	F_d
DW1							DW2						
**1**	0.28±0.01	0.35±0.01	0.55±0.03				**8**	0.03±0.00	0.07±0.00	0.06±0.01			
**2**	0.12±0.01	0.16±0.01	0.11±0.00				**9**	0.06±0.00	0.02±0.00	0.02±0.00			
**3**	0.12±0.01	0.11±0.03	0.22±0.01				**10**	0.03±0.01	0.02±0.01	0.07±0.00			
**4**	0.11±0.03	0.40±0.01	0.06±0.01				**11**	0.01±0.00	0.01±0.00	0.02±0.00			
**5**	0.69±0.00	0.97±0.01	0.92±0.01				**12**	0.02±0.00	0.02±0.00	0.01±0.00			
**6**	0.04±0.00	0.04±0.01	0.17±0.01				**13**	0.03±0.01	0.02±0.00	0.02±0.00			
**7**	0.08±.01	0.05±0.01	0.06±0.01				**14**	0.20±0.01	0.18±0.02	0.33±0.02			
**1,2,3,4**	0.35±0.02	0.47±0.02	0.58±0.02	1.23	1.18	1.07	**8,9,10,11**	0.02±0.00	0.02±0.00	0.04±0.00	0.37	0.23	0.62
**1,2,3,5**	0.57±0.02	0.66±0.04	0.78±0.01	0.83	0.69	0.84	**8,9,10,12**	0.03±0.01	0.02±0.01	0.03±0.00	0.40	0.23	0.44
**1,2,3,6**	0.56±0.00	0.61±0.02	0.73±0.02	1.98	1.75	1.34	**8,9,10,13**	0.03±0.00	0.02±0.00	0.04±0.00	0.47	0.28	0.50
**1,2,3,7**	0.37±0.01	0.41±0.02	0.53±0.02	1.29	1.19	0.97	**8,9,10,14**	0.06±0.01	0.11±0.03	0.17±0.01	0.97	0.65	0.52
**1,2,4,5**	0.61±0.01	0.84±0.05	0.89±0.02	0.88	0.87	0.97	**8,9,11,12**	0.02±0.00	0.03±0.00	0.02±0.00	0.31	0.37	0.35
**1,2,4,6**	0.56±0.02	0.44±0.02	0.48±0.01	1.97	1.10	0.88	**8,9,11,13**	0.02±0.01	0.03±0.00	0.02±0.00	0.34	0.37	0.42
**1,2,4,7**	0.47±0.02	0.35±0.01	0.32±0.01	1.65	0.88	0.59	**8,9,11,14**	0.07±0.00	0.13±0.01	0.18±0.00	0.34	0.76	0.55
**1,2,5,6**	0.89±0.02	0.91±0.02	0.91±0.03	1.29	0.94	0.99	**8,9,12,13**	0.02±0.00	0.02±0.00	0.01±0.01	0.24	0.24	0.25
**1,2,5,7**	0.66±0.02	0.70±0.02	0.90±0.01	0.95	0.73	0.98	**8,9,12,14**	0.08±0.01	0.13±0.01	0.18±0.01	0.38	0.75	0.53
**1,2,6,7**	0.49±0.03	0.49±0.01	0.70±0.01	1.74	1.42	1.28	**8,9,13,14**	0.11±0.01	0.11±0.01	0.21±0.01	0.55	0.64	0.63
**1,3,4,5**	0.48±0.03	0.66±0.03	0.89±0.02	0.70	0.68	0.96	**8,10,11,12**	0.04±0.01	0.02±0.00	0.05±0.01	1.06	0.21	0.76
**1,3,4,6**	0.54±0.03	0.54±0.02	0.60±0.03	1.90	1.38	1.10	**8,10,11,13**	0.02±0.00	0.01±0.00	0.04±0.01	0.51	0.18	0.55
**1,3,4,7**	0.33±0.02	0.47±0.01	0.58±0.01	1.16	1.18	1.06	**8,10,11,14**	0.11±0.01	0.10±0.02	0.23±0.01	0.52	0.55	0.70
**1,3,5,6**	0.55±0.01	0.88±0.02	0.71±0.02	0.79	0.91	0.77	**8,10,12,13**	0.01±0.00	0.01±0.00	0.03±0.00	0.19	0.17	0.42
**1,3,5,7**	0.63±0.02	0.72±0.02	0.78±0.05	0.92	0.74	0.84	**8,10,12,14**	0.16±0.01	0.15±0.01	0.25±0.00	0.79	0.86	0.76
**1,3,6,7**	0.53±0.01	0.57±0.01	0.71±0.04	1.88	1.66	1.30	**8,10,13,14**	0.17±0.01	0.18±0.02	0.31±0.02	0.84	1.02	0.93
**1,4,5,6**	0.88±0.01	0.80±0.06	0.81±0.02	1.28	0.82	0.87	**8,11,12,13**	0.01±0.00	0.01±0.00	0.04±0.00	0.29	0.13	0.63
**1,4,5,7**	0.67±0.05	0.76±0.06	0.88±0.06	0.97	0.79	0.95	**8,11,12,14**	0.13±0.02	0.15±0.02	0.32±0.01	0.66	0.88	0.96
**1,4,6,7**	0.62±0.03	0.41±0.02	0.53±0.03	2.17	1.03	0.97	**8,11,13,14**	0.14±0.01	0.12±0.00	0.30±0.01	0.69	0.69	0.91
**1,5,6,7**	0.57±0.02	0.94±0.04	0.91±0.05	0.83	0.97	0.98	**8,12,13,14**	0.13±0.00	0.12±0.00	0.26±0.00	0.66	0.70	0.79
**2,3,4,5**	0.51±0.02	0.52±0.01	0.82±0.03	0.73	0.54	0.88	**9,10,11,12**	0.02±0.00	0.04±0.01	0.06±0.01	0.39	1.93	1.81
**2,3,4,6**	0.89±0.02	0.50±0.04	0.79±0.05	7.14	1.25	3.58	**9,10,11,13**	0.04±0.01	0.04±0.01	0.07±0.01	0.58	2.41	2.12
**2,3,4,7**	0.39±0.03	0.36±0.02	0.67±0.01	3.10	0.91	3.04	**9,10,11,14**	0.12±0.02	0.15±0.02	0.13±0.02	0.61	0.87	0.40
**2,3,5,6**	1.05±0.03	0.77±0.07	0.97±0.02	1.51	0.79	1.05	**9,10,12,13**	0.03±0.00	0.05±0.00	0.09±0.02	0.48	2.50	2.58
**2,3,5,7**	0.57±0.00	0.51±0.02	0.71±0.07	0.83	0.53	0.76	**9,10,12,14**	0.12±0.01	0.16±0.02	0.17±0.02	0.59	0.91	0.51
**2,3,6,7**	0.79±0.01	0.53±0.02	0.81±0.06	6.35	3.31	3.66	**9,10,13,14**	0.14±0.01	0.16±0.02	0.18±0.02	0.70	0.90	0.54
**2,4,5,6**	0.85±0.06	0.70±0.06	0.87±0.04	1.23	0.72	0.94	**9,11,12,13**	0.02±0.01	0.02±0.01	0.16±0.03	0.33	1.20	6.43
**2,4,5,7**	0.62±0.04	0.55±0.01	0.61±0.03	0.89	0.57	0.66	**9,11,12,14**	0.10±0.01	0.17±0.04	0.04±0.00	0.51	0.96	0.13
**2,4,6,7**	0.28±0.01	0.13±0.01	0.22±0.01	2.24	0.33	1.29	**9,11,13,14**	0.13±0.01	0.09±0.02	0.20±0.02	0.66	0.53	0.61
**2,5,6,7**	0.76±0.03	0.94±0.03	0.90±0.05	1.09	0.97	0.97	**9,12,13,14**	0.12±0.01	0.08±0.02	0.16±0.02	0.59	0.43	0.49
**3,4,5,6**	0.84±0.06	0.86±0.02	1.00±0.02	1.21	0.89	1.08	**10,11,12,13**	0.02±0.00	0.05±0.03	0.02±0.00	2.17	2.47	0.72
**3,4,5,7**	0.47±0.04	0.71±0.03	0.74±0.02	0.68	0.73	0.80	**10,11,12,14**	0.26±0.02	0.13±0.04	0.34±0.01	1.28	0.76	1.03
**3,4,6,7**	0.84±0.03	0.65±0.02	0.72±0.00	7.25	1.64	3.28	**10,11,13,14**	0.27±0.03	0.10±0.01	0.40±0.02	1.35	0.57	1.19
**3,5,6,7**	0.82±0.04	0.88±0.04	0.88±0.01	1.19	0.90	0.96	**10,12,13,14**	0.26±0.01	0.15±0.05	0.40±0.01	1.31	0.86	1.21
**4,5,6,7**	0.83±0.11	0.66±0.03	0.74±0.02	1.19	0.68	0.80	**11,12,13,14**	0.29±0.01	0.12±0.02	0.42±0.03	1.44	0.68	1.27

Isolate	Biofilm formation (Abs590)			Trial 1	Trial 2	Trial 3	Isolate	Biofilm formation (Abs590)			Trial 1	Trial 2	Trial 3
	Trial 1	Trial 2	Trial 3	F_d	F_d	F_d		Trial 1	Trial 2	Trial 3	F_d	F_d	F_d
DW3							DW4						
**15**	0.06±0.01	0.16±0.03	0.04±0.00				**22**	0.19±0.04	0.13±0.00	0.19±0.02			
**16**	0.02±0.00	0.01±0.00	0.00±0.00				**23**	0.02±0.01	0.09±0.02	0.17±0.00			
**17**	0.07±0.00	0.02±0.00	0.01±0.01				**24**	0.04±0.01	0.07±0.01	0.09±0.00			
**18**	0.02±0.00	0.06±0.01	0.08±0.01				**25**	0.11±0.00	0.04±0.00	0.40±0.03			
**19**	0.01±0.00	0.20±0.01	0.01±0.00				**26**	0.00±0.01	0.00±0.00	0.05±0.00			
**20**	0.02±0.00	0.13±0.02	0.02±0.01				**27**	0.01±0.01	0.01±0.00	0.04±0.01			
**21**	0.33±0.02	0.03±0.00	0.00±0.00				**28**	0.18±0.01	0.13±0.01	0.10±0.00			
**15,16,17,18**	0.04±0.00	0.07±0.00	0.07±0.00	0.62	0.42	0.81	**22,23,24,25**	0.57±0.01	0.38±0.05	0.62±0.01	3.06	2.99	1.55
**15,16,17,19**	0.03±0.00	0.10±0.01	0.10±0.01	0.44	0.51	2.28	**22,23,24,26**	0.64±0.06	0.63±0.03	0.40±0.02	3.42	4.94	8.28
**15,16,17,20**	0.04±0.00	0.07±0.00	0.07±0.00	0.50	0.46	1.61	**22,23,24,27**	0.75±0.01	0.61±0.05	0.47±0.03	4.04	4.72	2.69
**15,16,17,21**	0.17±0.01	0.07±0.01	0.07±0.01	0.52	0.43	1.51	**22,23,24,28**	0.68±0.08	0.60±0.02	0.45±0.02	3.64	4.69	2.59
**15,16,18,19**	0.02±0.00	0.06±0.01	0.06±0.01	0.35	0.32	0.78	**22,23,25,26**	0.45±0.03	0.23±0.02	0.76±0.01	2.43	1.79	1.91
**15,16,18,20**	0.02±0.00	0.07±0.01	0.07±0.01	0.42	0.42	0.81	**22,23,25,27**	0.47±0.03	0.22±0.01	0.77±0.08	2.52	1.93	1.94
**15,16,18,21**	0.18±0.00	0.05±0.01	0.05±0.01	0.55	0.34	0.66	**22,23,25,28**	0.40±0.03	0.21±0.01	0.65±0.02	2.14	1.60	1.62
**15,16,19,20**	0.01±0.01	0.06±0.01	0.06±0.01	0.22	0.29	1.28	**22,23,26,27**	0.63±0.01	0.49±0.02	0.59±0.02	3.37	3.81	12.27
**15,16,19,21**	0.18±0.01	0.04±0.01	0.04±0.01	0.53	0.22	1.00	**22,23,26,28**	0.79±0.07	0.30±0.02	0.35±0.02	4.24	2.34	7.32
**15,16,20,21**	0.21±0.01	0.04±0.00	0.04±0.00	0.63	0.24	0.84	**22,23,27,28**	0.88±0.12	0.32±0.02	0.42±0.04	4.72	2.48	2.39
**15,17,18,19**	0.05±0.01	0.04±0.00	0.04±0.00	0.76	0.20	0.49	**22,24,25,26**	0.51±0.05	0.52±0.03	0.61±0.01	2.73	4.06	1.54
**15,17,18,20**	0.04±0.01	0.04±0.01	0.04±0.01	0.55	0.23	0.49	**22,24,25,27**	0.75±0.01	0.46±0.05	0.71±0.02	3.99	3.57	1.77
**15,17,18,21**	0.23±0.01	0.04±0.01	0.04±0.01	0.70	0.27	0.51	**22,24,25,28**	0.56±0.10	0.45±0.01	0.63±0.04	2.99	3.48	1.58
**15,17,19,20**	0.03±0.00	0.05±0.00	0.05±0.00	0.42	0.24	1.07	**22,24,26,27**	0.96±0.04	0.79±0.03	0.51±0.01	5.13	6.11	10.56
**15,17,19,21**	0.22±0.00	0.04±0.00	0.04±0.00	0.76	0.22	0.97	**22,24,26,28**	0.68±0.02	0.68±0.02	0.42±0.01	3.66	5.27	8.73
**15,17,20,21**	0.27±0.02	0.03±0.00	0.03±0.00	0.93	0.19	0.65	**22,24,27,28**	0.57±0.03	0.60±0.02	0.43±0.02	3.06	4.64	2.23
**15,18,19,20**	0.04±0.00	0.02±0.00	0.02±0.00	0.63	0.08	0.19	**22,25,26,27**	0.39±0.04	0.24±0.01	0.49±0.03	2.08	1.89	1.23
**15,18,19,21**	0.32±0.01	0.01±0.01	0.01±0.01	0.96	0.07	0.16	**22,25,26,28**	0.43±0.06	0.23±0.01	0.52±0.04	2.29	1.76	1.32
**15,18,20,21**	0.30±0.01	0.02±0.01	0.02±0.01	0.91	0.12	0.24	**22,25,27,28**	0.46±0.06	0.23±0.01	0.48±0.03	2.46	2.00	1.22
**15,19,20,21**	0.23±0.00	0.01±0.00	0.01±0.00	0.79	0.05	0.20	**22,26,27,28**	0.68±0.04	0.37±0.01	0.39±0.03	3.65	2.87	8.04
**16,17,18,19**	0.09±0.01	0.19±0.01	0.19±0.01	1.30	0.96	2.34	**23,24,25,26**	0.08±0.01	0.05±0.00	0.10±0.01	0.71	0.54	0.23
**16,17,18,20**	0.07±0.01	0.20±0.02	0.20±0.02	1.02	1.51	2.38	**23,24,25,27**	0.10±0.00	0.06±0.01	0.11±0.00	0.89	0.65	0.29
**16,17,18,21**	0.13±0.02	0.12±0.01	0.12±0.01	0.40	2.11	1.50	**23,24,25,28**	0.12±0.00	0.10±0.01	0.17±0.00	0.64	0.80	0.43
**16,17,19,20**	0.09±0.02	0.16±0.02	0.23±0.02	1.24	0.78	14.47	**23,24,26,27**	0.07±0.00	0.04±0.01	0.15±0.01	1.67	0.46	0.88
**16,17,19,21**	0.17±0.02	0.14±0.02	0.24±0.02	0.51	0.71	22.69	**23,24,26,28**	0.08±0.00	0.06±0.01	0.09±0.01	0.46	0.48	0.50
**16,17,20,21**	0.18±0.02	0.15±0.02	0.15±0.02	0.54	1.14	8.23	**23,24,27,28**	0.10±0.01	0.06±0.00	0.12±0.00	0.55	0.51	0.68
**16,18,19,20**	0.16±0.03	0.18±0.02	0.18±0.02	6.43	0.92	2.22	**23,25,26,27**	0.12±0.01	0.11±0.02	0.17±0.00	1.09	1.16	0.43
**16,18,19,21**	0.04±0.00	0.12±0.01	0.12±0.01	0.13	0.58	1.42	**23,25,26,28**	0.28±0.00	0.13±0.01	0.23±0.02	1.53	1.03	0.58
**16,18,20,21**	0.20±0.02	0.08±0.01	0.18±0.01	0.61	0.64	2.21	**23,25,27,28**	0.22±0.02	0.09±0.00	0.19±0.01	1.19	0.68	0.48
**16,19,20,21**	0.16±0.02	0.20±0.05	0.11±0.05	0.49	0.98	6.06	**23,26,27,28**	0.29±0.01	0.19±0.07	0.18±0.01	1.60	1.53	1.02
**17,18,19,20**	0.02±0.00	0.12±0.03	0.22±0.03	0.35	0.60	2.69	**24,25,26,27**	0.04±0.01	0.02±0.00	0.14±0.01	0.35	0.27	0.35
**17,18,19,21**	0.34±0.01	0.03±0.01	0.21±0.01	1.03	0.16	2.52	**24,25,26,28**	0.05±0.01	0.03±0.00	0.28±0.04	0.22	0.20	0.71
**17,18,20,21**	0.40±0.02	0.15±0.02	0.15±0.02	1.19	1.20	1.87	**24,25,27,28**	0.05±0.01	0.03±0.01	0.30±0.00	0.23	0.22	0.76
**17,19,20,21**	0.50±0.01	0.14±0.01	0.24±0.01	1.51	0.71	13.68	**24,26,27,28**	0.06±0.00	0.02±0.00	0.11±0.01	0.30	0.13	1.05
**18,19,20,21**	0.42±0.03	0.17±0.02	0.17±0.02	1.27	0.83	2.02	**25,26,27,28**	0.23±0.01	0.17±0.01	0.33±0.01	1.41	1.27	0.82

Table S3: Biofilm formed by single and combinations of four species isolated from sample DW4. Biofilm quantification was done after 24 and 120 hours of incubation at 25°C by crystal violet staining and absorbance measured at 590 nm. $\mathbf{F_{d1}}$ is the ratio of (Abs 590 multispecies bacterial biofilm – St Err) / (Abs 590 best single bacterial species + St Err) or (Abs 590 multispecies bacterial biofilm + St Err) / (Abs 590 best single bacterial species - St Err). $\mathbf{F_{d2}}$ is is the ratio of (Abs 590 multispecies bacterial-fungal biofilm – St Err) / (Abs 590 best single bacterial species co-cultured with fungi + St Err) or (Abs 590 multispecies bacterial-fungal biofilm + St Err) / (Abs 590 best single bacterial species co-cultured with fungi - St Err). $\mathbf{F_{d3} > 1}$ **(**$\mathbf{F_{d3} = F_{d2}/F_{d1}}$**)** determines the total biofilm induction in the presence of *E. dermatitidis* cells. The combinations 22, 23, 24, 27 (Consortia 1) and 22, 24, 25, 27 (Consortia 2) had $\mathbf{F_{d3}}$ value > 1 in all the three biological trails signifying an increase in the biofilm production due to the addition of *E. dermatitidis* cells. These two consortia were further investigated for bacterial and fungal cell number quantification and biofilm formation on EPDM and PP materials.

Isolate	Biofilm formation (Abs590)			Biofilm formation in the presence of *E. dermatitidis* (Abs590)			F_{d1}	F_{d2}	F_{d3}	F_{d1}	F_{d2}	F_{d3}	F_{d1}	F_{d2}	F_{d3}
	Trial 1	Trial 2	Trial 3	Trial 1	Trial 2	Trial 3	Trial 1			Trial 2			Trial 3		
**24 hours**															
**22**	0.056±0.007	0.014±0.000	0.047±0.003	0.079±0.003	0.038±0.000	0.091±0.002	-	-	-	-	-	-	-	-	-
**23**	0.008±0.002	0.001±0.002	0.012±0.000	0.044±0.010	0.010±0.003	0.047±0.010	-	-	-	-	-	-	-	-	-
**24**	0.024±0.001	0.031±0.001	0.003±0.003	0.022±0.018	0.025±0.011	0.021±0.000	-	-	-	-	-	-	-	-	-
**25**	0.017±0.001	0.015±0.002	0.031±0.001	0.031±0.018	0.018±0.024	0.026±0.018	-	-	-	-	-	-	-	-	-
**26**	0.008±0.003	0.018±0.000	0.009±0.001	0.004±0.003	0.010±0.024	0.070±0.027	-	-	-	-	-	-	-	-	-
**27**	0.006±0.003	0.007±0.002	0.002±0.002	0.002±0.005	0.000±0.023	0.103±0.040	-	-	-	-	-	-	-	-	-
**28**	0.070±0.010	0.009±0.001	0.036±0.000	0.072±0.005	0.053±0.021	0.074±0.007	-	-	-	-	-	-	-	-	-
**22,23,24,26**	0.236±0.012	0.175±0.004	0.149±0.005	0.289±0.005	0.320±0.007	0.324±0.010	4.23	3.66	0.86	5.61	8.36	1.49	3.15	3.56	1.13
**22,23,24,27**	0.481±0.014	0.204±0.006	0.158±0.005	0.701±0.021	0.353±0.010	0.386±0.018	8.62	8.87	1.03	6.55	9.22	1.41	3.35	8.14	2.43
**22,23,24,28**	0.400±0.029	0.148±0.006	0.186±0.006	0.557±0.011	0.272±0.006	0.300±0.016	7.18	7.04	0.98	4.74	5.09	1.07	3.94	14.00	3.55
**22,23,26,27**	0.562±0.049	0.125±0.001	0.195±0.004	0.703±0.016	0.230±0.001	0.380±0.022	10.07	8.90	0.88	7.06	6.02	0.85	4.13	14.61	3.54
**22,24,25,27**	0.421±0.008	0.158±0.005	0.214±0.009	0.737±0.048	0.260±0.015	0.481±0.024	7.55	9.33	1.23	5.05	6.81	1.35	4.53	6.85	1.51
**22,24,25,28**	0.194±0.009	0.128±0.006	0.131±0.007	0.202±0.008	0.284±0.000	0.184±0.013	3.47	2.56	0.74	4.10	5.33	1.30	2.77	1.79	0.65
**22,24,26,27**	0.234±0.028	0.250±0.009	0.225±0.022	0.269±0.014	0.311±0.017	0.362±0.016	4.20	3.40	0.81	8.02	8.12	1.01	4.77	4.86	1.02
**22,24,27,28**	0.162±0.029	0.178±0.006	0.126±0.026	0.210±0.018	0.284±0.006	0.327±0.014	2.90	2.66	0.92	5.69	5.33	0.94	2.67	1.01	0.38
**120 hours**															
**22**	0.160±0.007	0.150±0.004	0.157±0.004	0.222±0.002	0.136±0.009	0.129±0.003	-	-	-	-	-	-	-	-	-
**23**	0.213±0.009	0.021±0.001	0.015±0.001	0.288±0.010	0.040±0.006	0.307±0.047	-	-	-	-	-	-	-	-	-
**24**	0.098±0.009	0.057±0.052	0.002±0.001	0.135±0.020	0.006±0.014	0.024±0.005	-	-	-	-	-	-	-	-	-
**25**	0.022±0.001	0.019±0.011	0.027±0.005	0.009±0.023	0.028±0.011	0.001±0.002	-	-	-	-	-	-	-	-	-
**26**	0.007±0.001	0.003±0.001	0.018±0.004	0.007±0.015	0.051±0.028	0.034±0.004	-	-	-	-	-	-	-	-	-
**27**	0.015±0.003	0.001±0.002	0.009±0.001	0.005±0.022	0.013±0.008	0.019±0.008	-	-	-	-	-	-	-	-	-
**28**	0.067±0.003	0.025±0.002	0.034±0.001	0.109±0.023	0.026±0.001	0.038±0.002	-	-	-	-	-	-	-	-	-
**22,23,24,26**	0.454±0.031	0.321±0.073	0.785±0.032	0.776±0.007	0.487±0.015	0.775±0.017	2.13	2.70	1.27	2.15	3.58	1.67	5.01	2.52	0.50
**22,23,24,27**	0.334±0.026	0.372±0.085	0.284±0.024	0.777±0.018	0.628±0.022	0.931±0.015	1.56	2.70	1.73	2.48	4.62	1.86	1.81	3.03	1.67
**22,23,24,28**	0.335±0.027	0.514±0.028	0.559±0.004	0.674±0.029	0.560±0.004	0.601±0.012	1.57	2.34	1.49	3.43	4.12	1.20	3.57	1.96	0.55
**22,23,26,27**	0.468±0.018	0.286±0.029	0.252±0.006	0.533±0.021	0.369±0.027	0.398±0.018	2.20	1.85	0.84	1.91	2.72	1.42	1.61	1.29	0.81
**22,24,25,27**	0.280±0.029	0.197±0.010	0.165±0.006	0.530±0.009	0.255±0.021	0.353±0.009	1.75	2.39	1.36	1.32	1.87	1.42	1.05	2.73	2.59
**22,24,25,28**	0.204±0.007	0.152±0.017	0.209±0.007	0.446±0.009	0.292±0.040	0.499±0.050	1.28	2.01	1.58	1.02	2.15	2.11	1.33	3.85	2.89
**22,24,26,27**	0.318±0.047	0.184±0.016	0.177±0.003	0.572±0.016	0.306±0.015	0.310±0.020	1.99	2.58	1.30	1.23	2.25	1.83	1.13	2.40	2.12
**22,24,27,28**	0.235±0.017	0.177±0.016	0.200±0.021	0.421±0.014	0.250±0.003	0.287±0.030	1.47	1.90	1.29	1.18	1.84	1.56	1.27	2.22	1.74
